# Delivering Clinical impacts of the MRI diagnostic pathway in prostate cancer diagnosis

**DOI:** 10.1007/s00261-020-02547-x

**Published:** 2020-04-30

**Authors:** Ivo G. Schoots, Anwar R. Padhani

**Affiliations:** 1 Department of Radiology & Nuclear Medicine, Erasmus MC University Medical Center, ’s-Gravendijkwal 230, P.O. Box 2040, 3000 CA Rotterdam, The Netherlands; 2 Department of Radiology, Netherlands Cancer Institute, Amsterdam, The Netherlands; 3 Paul Strickland Scanner Centre, Mount Vernon Cancer Centre, Northwood, UK

**Keywords:** Prostate cancer, Biopsy, Magnetic resonance imaging (MRI), Risk stratification, Multivariate risk prediction, Risk calculator, Nomogram

## Abstract

Pre-biopsy multiparametric MRI is now recommended by multiple guidelines, not only for men with persistent suspicion of prostate cancer after prior negative systematic biopsy, but also at initial screening before the first biopsy. The major benefit of pre-biopsy MRI in the diagnostic work-up is to promote individualized risk-adapted approaches for biopsy-decision management. Multiple MRI-directed diagnostic pathways can be conceived, with each approach having net-benefit trade-offs between benefits and harms, based on improved diagnostic yields of significant cancers and reduced biopsy testing and reduced detection of indolent prostate cancer. In this paper, we illustrate how clinical benefits can be maximized in men with MRI-negative and MRI-positive results, using the PI-RADS Multiparametric MRI and MRI-directed biopsy pathway. From a practice perspective, we emphasize five golden rules: (1) that multiparametric MRI approach including targeted biopsies be reserved for men likely to benefit from early detection and treatment of prostate cancer; (2) that there is a need to carefully assess risk of significant disease using PSA and clinical parameters before and after MRI; (3) do not offer immediate biopsy if the MRI is negative, unless other high-risk factors are present; (4) accept that not all significant cancers are found immediately and have robust ‘safety nets’ for men with negative MRI scans who avoid immediate biopsy and for positive MRI patients with negative or non-explanatory histology; and (5) use MRI-directed biopsy methods that minimize overdiagnosis and improve risk stratification.

## Introduction

The Prostate Imaging Reporting and Data System (PI-RADS) MRI-directed biopsy pathway [1] enables the delivery of multiple diagnostic and clinical benefits to men suspected of having significant cancer according to the National Comprehensive Cancer Network (NCCN), American Urological Association (AUA) and the European Association of Urology (EAU) [2–4]. Thereby, both biopsy-naive men and men at continued clinical suspicion after a negative systematic biopsy, with a higher than average risk of prostate cancer, may undergo MRI before prostate biopsy. We intent to clarify the earlier proposed MRI risk-adapted approach using PI-RADS assessment for the need for biopsy [1].

## MRI-directed pathway

When prostate MRI is used to detect significant disease, several MRI-directed biopsy strategies can be adopted, each having different impacts on the number of needle cores used and on the detection rates of significant and insignificant prostate cancers [5]. MRI results can be used in two distinct ways. The first is the combined biopsy pathway, in which patients with negative MRI findings undergo systematic biopsy and those with positive MRI findings undergo both systematic and MRI-directed biopsy, thus maximizing the diagnostic yield of clinically significant cancers [1, 4, 5]. The second is the MRI pathway, which is distinct in that men with negative MRI findings do not undergo biopsy at all and men with positive findings undergo only MRI-directed biopsy (without systematic cores), thus minimizing the diagnosis of indolent disease. Several other MRI-directed biopsy combinations can also be envisioned each of which balances the detection of significant and insignificant prostate cancers [5].

## Clinical benefits

When determining the clinical utility of prostate cancer diagnosis, it is important to identify only those men that are likely to benefit from timely diagnoses. Clinical benefit is therefore the detection and appropriate treatment of clinically significant prostate cancer. Clinical harms include redundant (unnecessary) testing and the likelihood of having complications of testing (such as rectal bleeding, urine retention, bacteremia and urosepsis). Remembering that many of the cancers detected may never become clinically evident; therefore, overdiagnoses and subsequent over-treatments can also contribute to harms.

In the prostate cancer diagnostic work-up of biopsy-naïve men, the main advantage of the MRI pathway is to reduce the number of men who need biopsies and to reduce the total number of biopsy cores in men with positive MRI findings, thus helping to limit overdiagnoses of clinically insignificant disease [6–8]. Furthermore, MRI-directed biopsies improve tumor grade classifications, tumor volume estimations and subsequently improve patient risk assessments [9], all of which are important for the accurate guidance of treatment decisions.

## Patient selection for MRI

Patient selection criteria for MRI prior to biopsy are not clear because of limited inclusion criteria within clinical studies that often exclude men with low and very high risk of having clinically significant cancers. Few studies have evaluated MRI benefits by clinical inclusion criteria beyond looking at biopsy naïve versus prior negative biopsy cases. Even in these groups, there are inconsistencies between the NCCN, AUA and EAU guidelines regarding the use of MRI. The only conclusion that can be drawn is that MRI should be used whenever a prostate biopsy is indicated (Table 1).

**Table 1:** Key guidance and statements for the use of MRI for prostate cancer diagnosis

Statement	Organization
Guidance for early prostate cancer detection in asymptomatic men	
Do not subject men to prostate-specific antigen (PSA) testing without counseling them on the potential risks and benefits	EAU 2020
Offer an individualized risk-adapted strategy for early detection to a well-informed man with a good performance status and a life expectancy of at least 10 to 15 years	EAU 2020, AUA 2019, NCCN 2019
Offer early PSA testing in well-informed men at elevated risk of having prostate cancer (> 50 years of age, > 45 years of age and family history of prostate cancer, African descent, BRCA2 mutations carriers)	EAU 2020
Guidance for MRI use for diagnosing prostate cancer	
Do not use MRI as an initial screening tool for unselected men	EAU 2020 PI-RADS 2019
Adhere to PI-RADS guidelines for multiparametric magnetic resonance imaging (mpMRI) acquisition and interpretation and evaluate mpMRI results in multidisciplinary meetings with pathological feedback	EAU 2020 PI-RADS 2019
Carefully assess/reassess risk of significant disease using PSA metrics and/or risk calculators before and after MRI; combine clinical parameters and MRI results for deciding biopsy need	Current manuscript
Guidance in biopsy naïve men	
With negative MRI scans at low suspicion	
Omit biopsy based on shared decision making	EAU 2020 PI-RADS 2019
Have robust ‘safety net’ for men who avoid immediate biopsy with roles and responsibility clearly defined	PI-RADS 2019
Discharge patients to primary care if the level of suspicion is low advise PSA follow-up at 6 months and then every year set PSA level at which to re-refer based on PSAD (0.15 ng/ml/ml) or velocity (0.75 ng/year)	NICE 2019
With negative MRI scans at high suspicion	
Perform systematic biopsy based on shared decision	EAU 2020
Offer prostate biopsy if there is a strong suspicion of prostate cancer based on PSA density (> 0.15 ng/ml/ml) or PSA velocity (> 0.75 ng/year), or strong family history, taking into account life expectancy and comorbidities	NICE 2019
With positive MRI scans	
Use MRI as a roadmap to guide biopsy procedures to increase precision of biopsy	PI-RADS 2019
Combine systematic and targeted biopsy focal saturation is a viable alternative	EAU 2019 PI-RADS 2019
In men with prior negative biopsies, when MRI is positive perform targeted biopsy only	EAU 2019
Multiple re-biopsy options exist in men with negative or non-explanatory histology after MRI-directed biopsy at persistent high risk	PI-RADS 2019
Guidance in prior negative biopsy men with persistent high suspicion	
Perform MRI before biopsy	EAU 2020, AUA 2019, NCCN 2019
When MRI is positive perform targeted biopsy only	EAU 2020
When MRI is negative, perform systematic biopsy based on shared decision making with the patient	EAU 2020
Multiple re-biopsy options exist in men with negative or non-explanatory histology after MRI-directed biopsy at persistent high risk	PI-RADS 2019

*PSA* prostate-specific antigen, *MRI* magnetic resonance imaging, *PI-RADS* Prostate Imaging Reporting and Data System, *NCCN* National Comprehensive Cancer Network, *AUA* American Urology Association, *EAU* European Association of Urology, *NICE* UK National Institute of Clinical Care Excellence

**Fig. 1:**
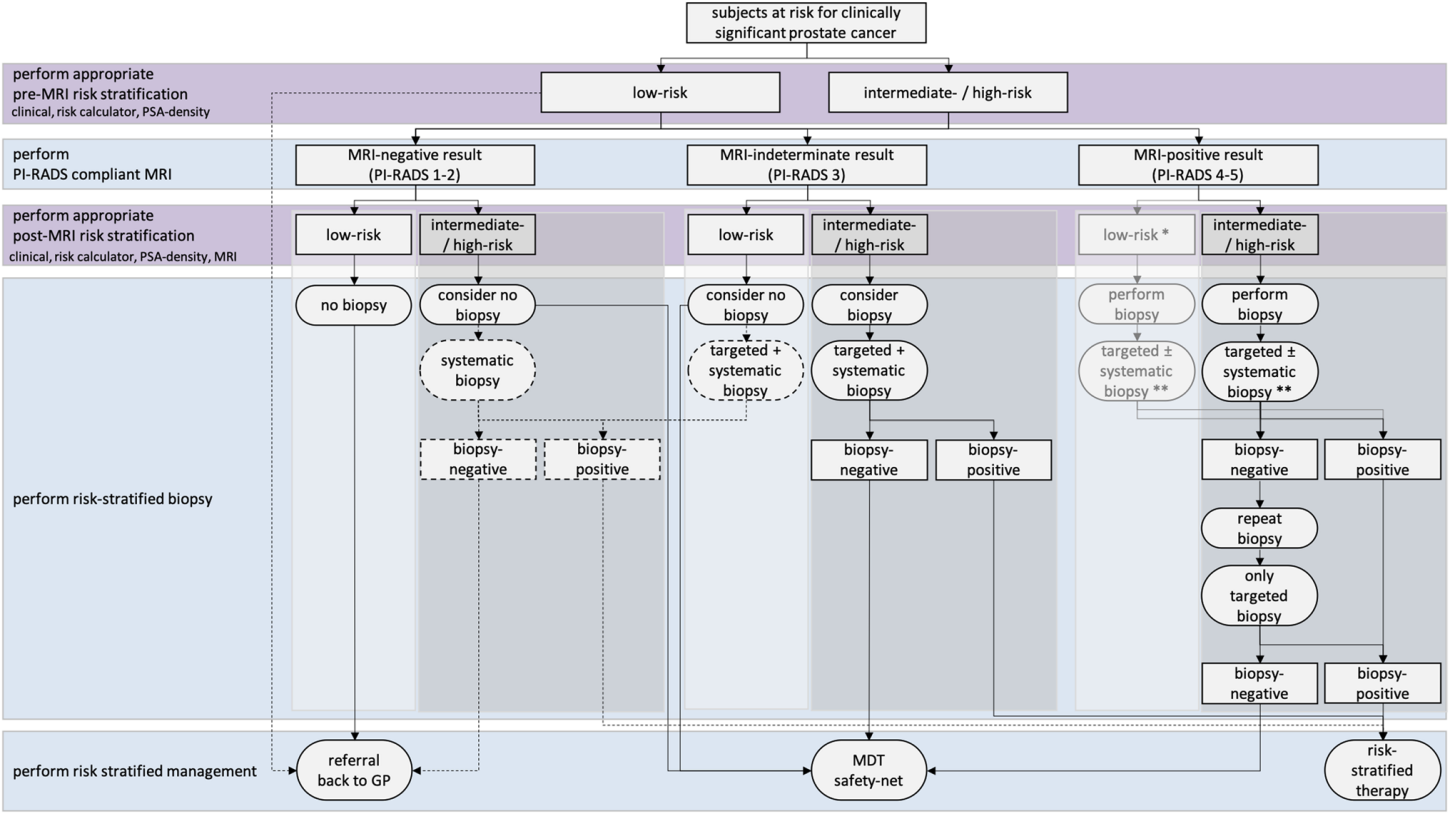
Flowchart of MRI pathway including patient risk stratification, when to perform biopsy. On the left suggested pre- and post-MRI risk stratification (purple) and related actions (blue), on the right the flowchart with actions (ovals) and results (rectangles). Identification of low-risk (light gray boxes) and intermediate-/high-risk men (darker gray boxes). Pathway preference (continuous lines) and alternative routes (dashed lines) are combined. *This route is more theoretical: a man with a PI-RADS 4 or 5 score at first diagnosis most likely will not be categorized in low risk, only after negative biopsies despite consistent PI-RADS 4 or 5 scores, as a results of MRI false positives. **This route with targeted and systematic biopsies can further be adapted to specific clinical scenarios. A man with a large PI-RADS 5 focus may already benefit from only MRI-directed targeted biopsy. A man with a small PI-RADS 4 focus may benefit from only MRI-directed focal saturation biopsy, sampling the suspected region and its penumbra. *PSA* prostate-specific antigen, *MRI* magnetic resonance imaging, *GP* general practitioner, *MDT* multidisciplinary team

Patient risk stratification is used in clinical practice to reduce unnecessary biopsies (and by extension the need for MR imaging) [4]. Clinical biopsy decisions are based on clinical factors such as serum PSA levels, rectal examination findings, prostate volume estimates, age and family history. Although the decision to biopsy is based on individual preferences, there is a need to balance harms (overdiagnosis, biopsy-related) and benefits (diagnosis and therapy of significant disease). As a general guideline, the EAU guideline advises that to avoid unnecessary biopsies, further risk assessments be undertaken of asymptomatic men with a normal digital rectal examination and a PSA level between 2 and 10 ng/mL prior to performing a prostate biopsy. These further risk assessments can include MRI (Fig. 1).

A major advantage of MRI is that it enables independent assessments of the likelihood of clinically significant disease based on image only observations. The likelihood of clinically significant cancer can be low (PI-RADS category 1 or 2), intermediate (PI-RADS category 3) or high (PI-RADS category 4 or 5) [10]. Men having an intermediate or high likelihood of having clinically significant cancer are regarded as having a ‘positive’ MRI, requiring prostate biopsies [1, 2, 4]. Men with negative MRI are stratified to a lower risk of having significant disease, a prostate biopsy can be considered depending on clinical risks, clinical priorities and patient preferences (Fig. 1) [1, 4].

However, MRI interpretations and the need for biopsy afterwards should also be in the context of patient care priorities, assessed by multidisciplinary teams. In general, in biopsy-naive men, there is an urgent need to minimize overdiagnosis, especially in lower-risk men. Of all biopsy-naïve men diagnosed with prostate cancer using the restricted selection criteria above, almost half are diagnosed with clinically insignificant prostate cancer [6] and opt for active surveillance. This proportion is too high and may be even higher in low-risk men undergoing MRI assessments. In other words, there continues to be overdiagnosis as we attempt to improve diagnosis of significant cancers. Therefore, MRI is a step-forward but is not a perfect test for detecting only significant cancers.

## When MRI is negative

As already noted, PI-RADS scores provide independent risk assessments complimentary to clinical multivariate risk calculators [11]. Because of the high negative predictive value of PI-RADS–compliant MRI protocols [12, 13], a high proportion of men can avoid immediate biopsy after negative MRI findings without substantially affecting the detection rates of clinically significant cancers (Fig. 1).

The yields of ISUP grade 2 prostate cancers or higher (Gleason score ≥ 3 + 4) on systematic biopsy after negative MRI in biopsy-naïve men is about 8%; the majority are (microfocal) ISUP grade 2 cancers [6]. Microfocal ISUP grade 2 cancers are prognostically good [14, 15]. When treated, prognosis of these ISUP grade 2 cancers is similar to ISUP grade 1 prostate cancer, when cribriform growth is absent [16]. A negative prostate MRI misses few ISUP grade 3 cancers or higher (Gleason score ≥ 4 + 3) that are detected by immediate systematic biopsy (~ 3%) [6, 12].

**Fig. 2:**
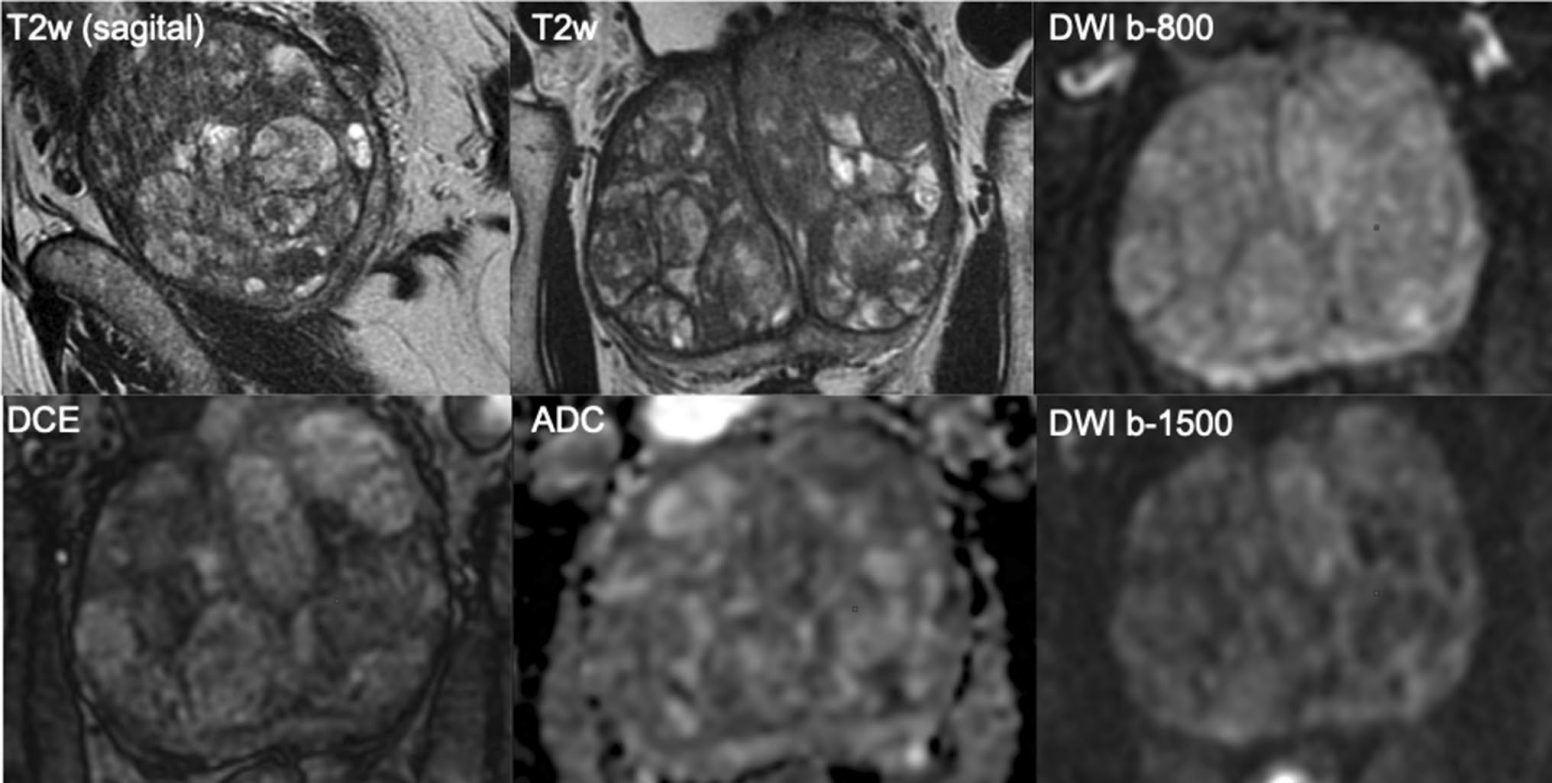
A 69-year-old man with a PSA of 42 ng/ml. Digital rectal examination (DRE) was normal, and transrectal ultrasound (TRUS) showed a prostate volume of 231 ml, without any suspected hypo-echoic lesions found. The PSA density was 0.18 ng/ml^2^. The need to perform MRI and biopsy: This man’s risk cannot be estimated by using current risk calculators, due to prostate volume limitations (max. prostate volume of 110 cc). While PSA was very high, the PSA density was in the range of intermediate risk. A low pre-test probability would be expected. The MRI was negative (PI-RADS v2 score 2). Histopathology biopsy findings and treatment management: Systematic biopsies revealed no prostate cancer. This man is in the institutional follow-up protocol. Considerations: Despite the suggested high risk due to high PSA levels, men with large prostate volumes as a result of BPH are less likely to harbor significant cancers. For these men, systematic biopsies should be avoided to minimize overdiagnoses after a negative MRI

**Fig. 3:**
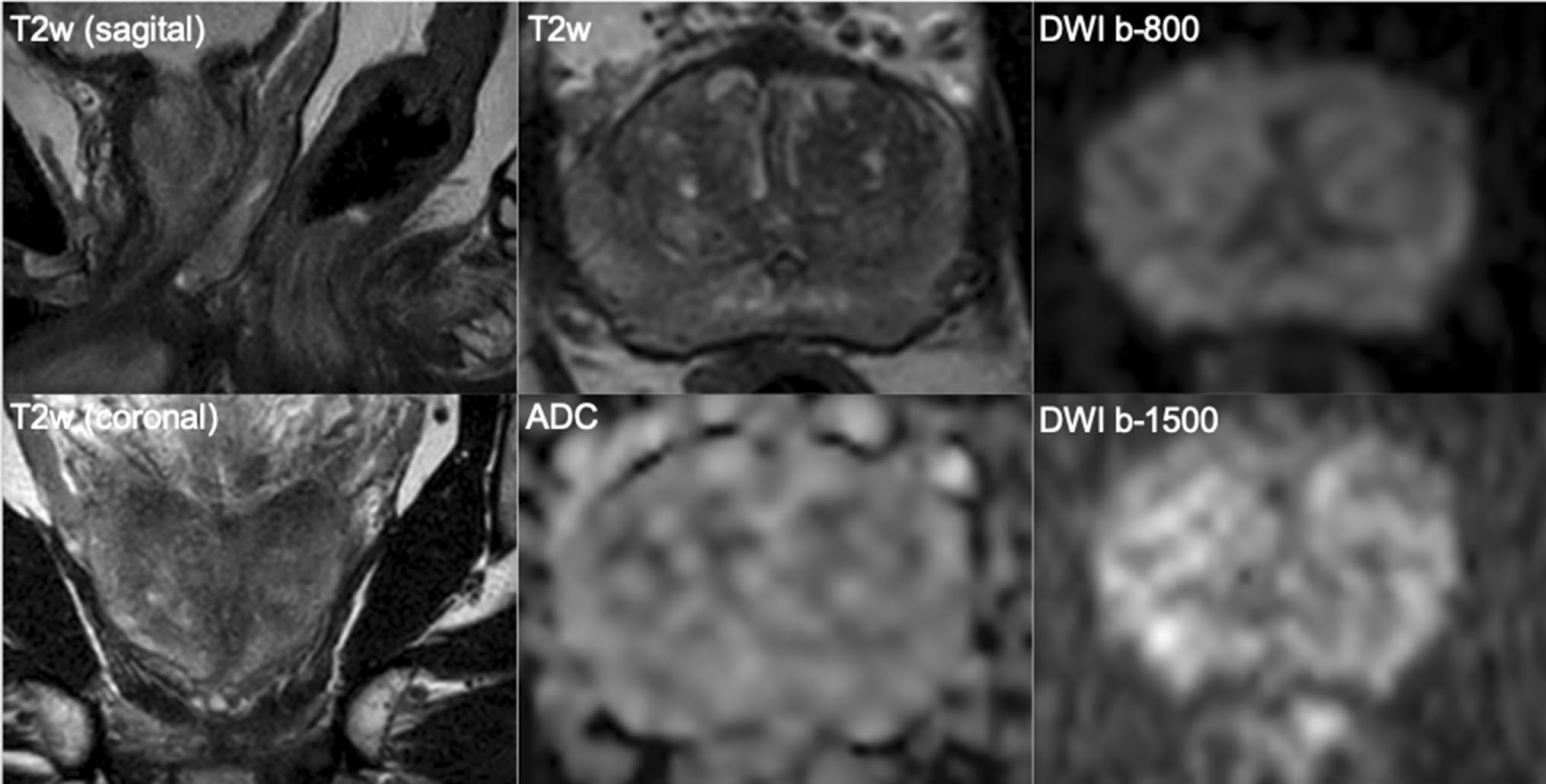
A 69-year-old man with a PSA of 6.9 ng/ml. Digital rectal examination (DRE) was normal, and transrectal ultrasound (TRUS) showed a prostate volume of 41 ml, without any hypo-echoic lesions found. The PSA density was 0.17 ng/ml^2^. The need to perform MRI and biopsy: Based on PSA and PSA density, a biopsy and a pre-biopsy MRI are both indicated. The MRI was negative (PI-RADS v2 score 2). Histopathology biopsy findings and treatment management: Systematic biopsies revealed no prostate cancer, only granulomatous inflammation on both sides. This man is in the institutional follow-up protocol. Considerations: In a man with a negative MRI with intermediate risk on clinical and PSA-density (≤ 0.15–0.20 ng/ml^2^) findings, deferral of immediate systematic biopsies can be considered with appropriate safety net protocol. Inflammation is a common cause of elevated serum PSA levels

**Fig. 4:**
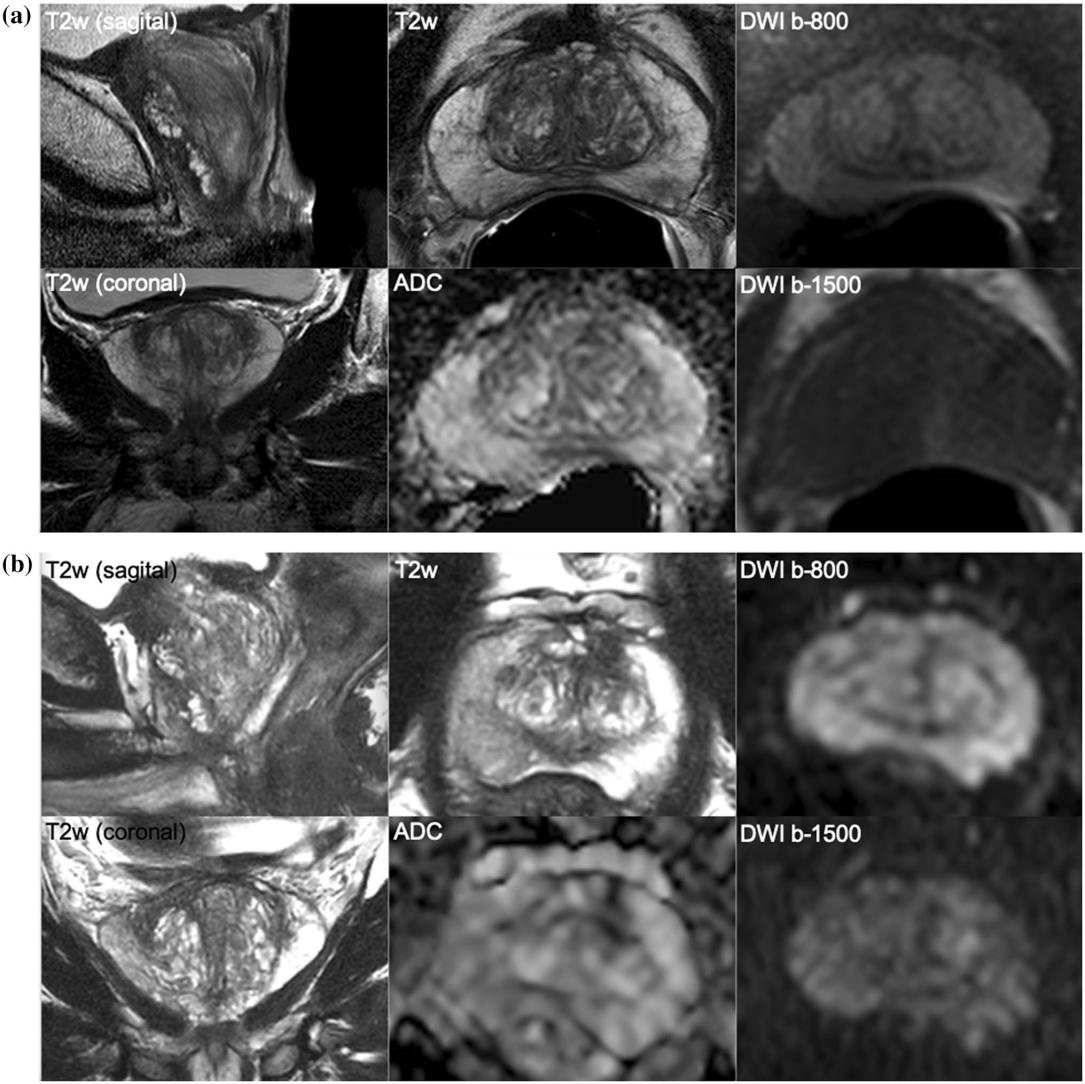
**a** and **b** A 68-year-old man with a PSA of 17.2 ng/ml. Digital rectal examination (DRE) was normal, and transrectal ultrasound (TRUS) showed a prostate volume of 40 ml, without any hypo-echoic lesions found. The PSA density was 0.43 ng/ml^2^. The need to perform MRI and biopsy: Based on the PSA and PSA density, a biopsy and a pre-biopsy MRI are both indicated. However, based on the negative MRI findings a systematic biopsy should be discussed given the very elevated PSA density (≥ 0.20 ng/ml^2^). Histopathology biopsy findings and treatment: This man underwent systematic biopsies without cancer detection, and then urological follow-up. Follow-up management: At 4 years of follow-up, the PSA increased to 28.1 ng/ml, without DRE suspicion or volume change. Based on PSA and PSA density, a biopsy and a pre-biopsy MRI were again indicated, despite previous negative biopsies. Again, the MRI findings were negative, and a systematic biopsy again discussed, due to the elevated PSA density. Histopathology biopsy findings and treatment management: Systematic biopsies revealed one positive core out of seven left-sided cores, with a Gleason score of 3 + 3. Right-sided cores were negative for cancer. Patient preferred active surveillance outside protocol (high risk owing to single risk factor—PSA levels), instead of active treatment. Considerations: No significant cancer has been detected, despite very high levels of PSA. When the PSA level is high, the avoidance of systematic biopsies in MRI-negative men is considered unsafe as some significant cancers (mostly Gleason 3 + 4) can be present. A urological safety net should be in place for men avoiding immediate systematic biopsy after a negative MRI scan who remain at high suspicion

A negative MRI strongly modulates likelihood of significant cancer and the need for biopsy, especially in men with large prostate gland volumes (Fig. 2). Furthermore, a negative MRI for prostate cancer can provide alternative explanations for raised PSA level, such as inflammation (Fig. 3). Yields of ISUP grade 1 prostate cancers (Gleason score = 3 + 3) on systematic biopsy after negative MRI in biopsy-naïve men (Fig. 4) is about 17%, which contributes significantly to overdiagnosis and overtreatment [6]. That is to say that for every man diagnosed with significant cancer by systematic biopsy after a negative MRI, there will be 2 men overdiagnosed with indolent disease [6]. To minimize overdiagnoses in MRI-negative men, biopsy rates should be minimized, reserving biopsies for men at higher clinical risk despite a negative MRI (Table 1).

The majority of emerging significant cancers in MRI-negative men appear within the first 2–3 years (Fig. 4) [17, 18]. Therefore, a monitoring safety net must be in place for patients who prefer deferring immediate biopsy after a negative MRI examination. The safety net should include periodic clinical examinations, laboratory assays and imaging, as per local clinical practices and be consistent with clinical goals for individual patients (Table 1) [1]. In such clinical circumstances, the clinical priorities, the roles and responsibilities of the participants, the underlying risks, and the circumstances that should trigger reinvestigations should all be clearly defined when counseling patients [4, 19].

## When MRI is positive

Yields of ISUP grade 2 prostate cancers or higher (Gleason score ≥ 3 + 4) on targeted and systematic biopsy after a positive MRI in biopsy-naïve men is about 44%, showing the population enrichment with significant disease, and therefore the substantial value of MRI in patient risk assessments [6]. Using MRI as a roadmap to guide biopsy procedures also increases the detection of significant cancers. The cancer detection rate of significant disease is 1.12 (95% CI 1.01–1.23) times better for the MRI pathway than for the systematic biopsy alone, representing a significant 12% increase by the MRI pathway for patients in mixed urological settings. The maximum yield in MRI-positive men is obtained by combining both targeted and systemic biopsy procedures, which is currently recommended in prostate cancer guidelines [2–4, 19]. Utilizing the augmented MRI pathway (including systematic cores) increases significant cancer detection, but compromises on overdiagnoses, while the MRI pathway only (no systematic cores) minimizes the detection of insignificant cancers [5].

**Fig. 5:**
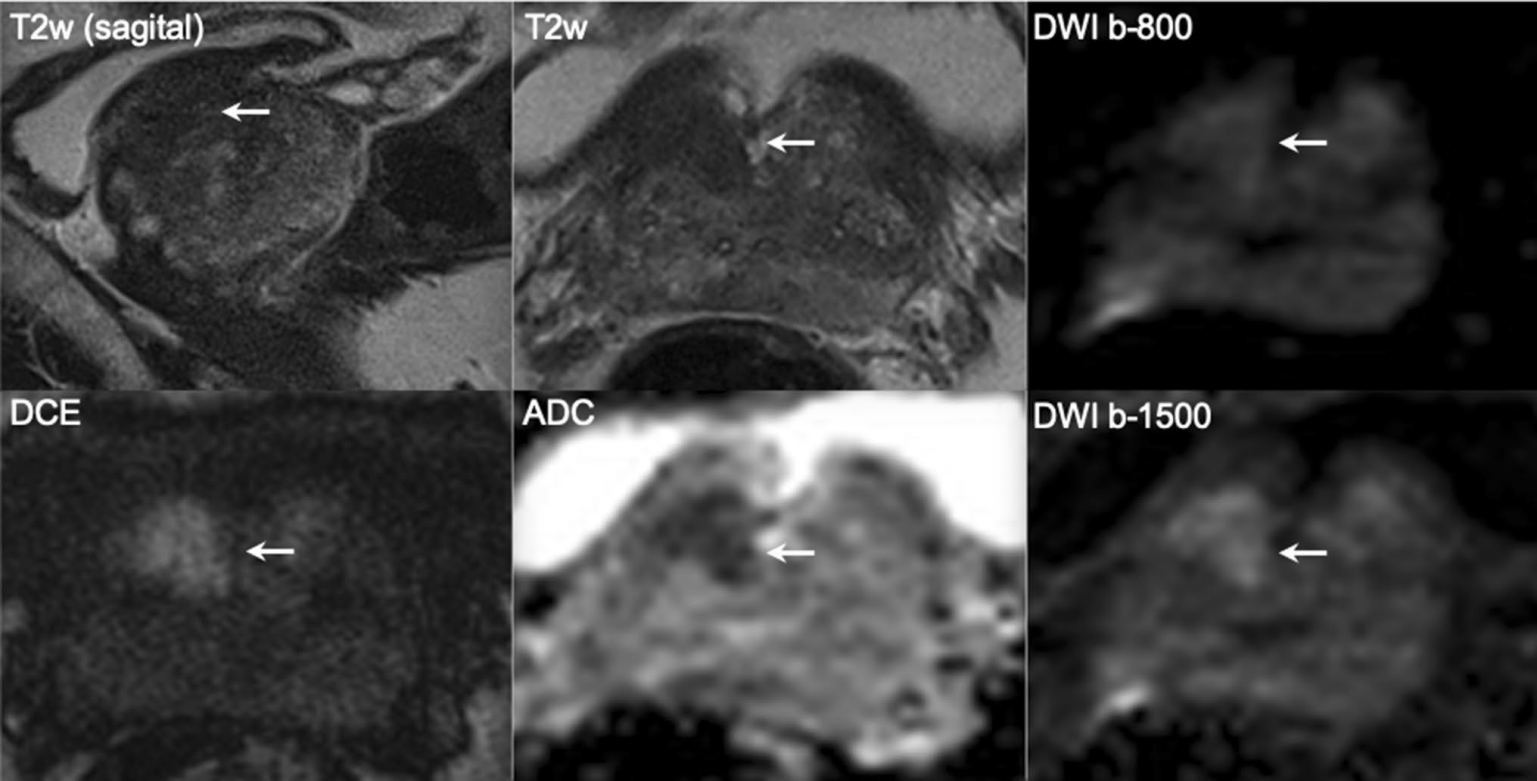
A 65-year-old man with a PSA of 10.6 ng/ml. Digital rectal examination (DRE) was normal, and transrectal ultrasound (TRUS) showed a prostate volume of 49 ml, without any hypo-echoic lesions found. The PSA density was 0.22 ng/ml^2^. The need to perform MRI and biopsy: Based on PSA, PSA density, a biopsy and a pre-biopsy MRI are both indicated. MRI identified a suspicious lesion of approximately 15 mm in the right transition zone, with substantial low ADC values and focal enhancement, without extraprostatic extension but with close proximity to the bladder neck. The total PI-RADS assessment category score was 5 (5/5/ +). Histopathology biopsy findings and treatment management: Biopsies revealed two positive cores out of three right-sided targeted cores with GS 3 + 3, without any cancer detection in five left-sided and five right-sided systematic biopsy cores. Patient preferred active surveillance outside clinical protocol (intermediate risk due to elevated PSA level > 10 ng/ml with visible tumor), instead of active treatment. Considerations: Use MRI findings as a roadmap to direct targeted biopsies. Large transition zone tumors may sometimes look very suspicious on imaging (PI-RADS 5) that on histologic evaluations have a GS 3 + 3 or GS 3 + 4. The clinical significance of these lesions is highly dependent on urologic preferences which should be understood by radiologists working in multidisciplinary teams

**Fig. 6:**
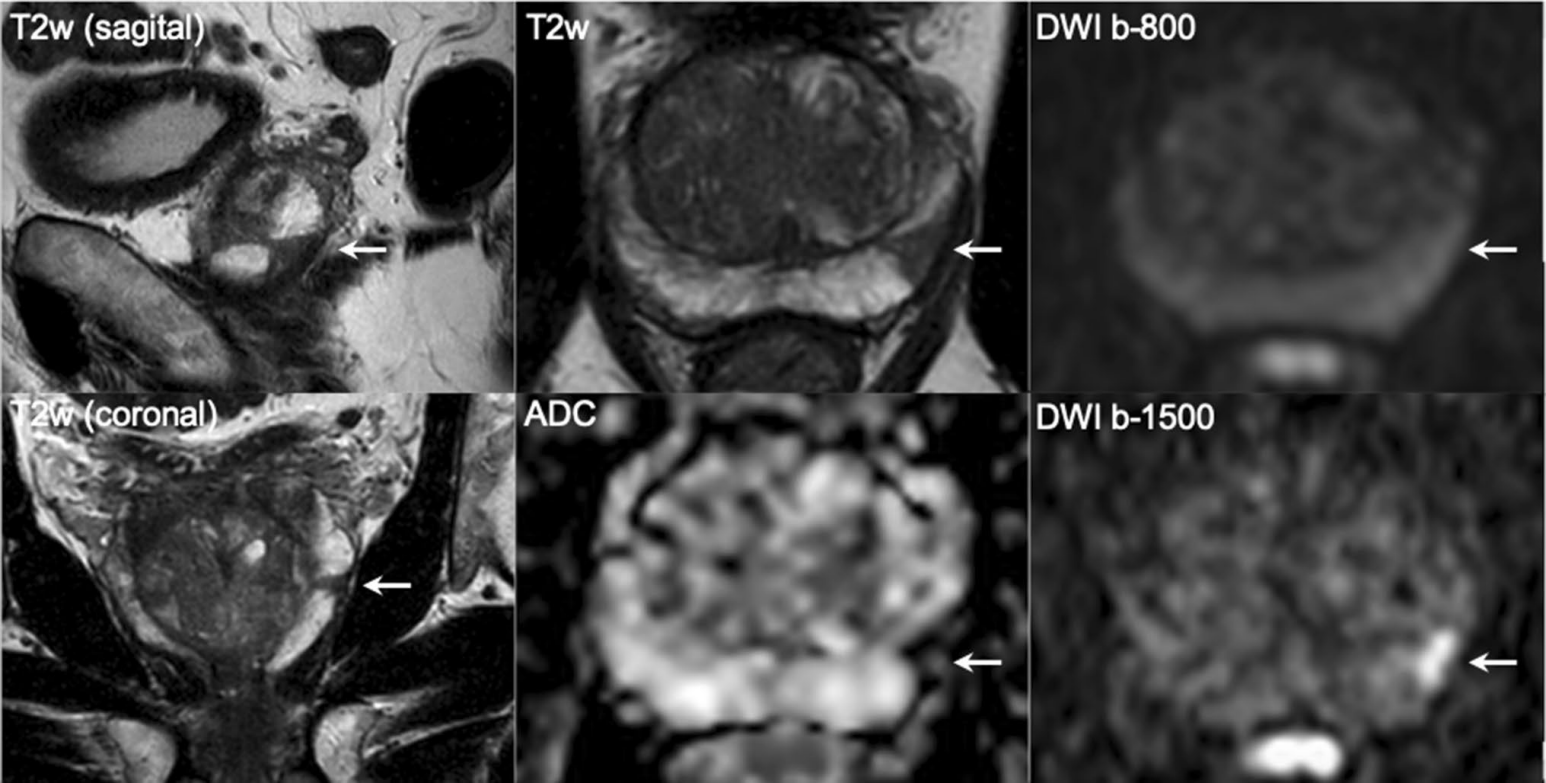
A 70-year-old man with a PSA of 11.1 ng/ml. Digital rectal examination (DRE) was normal, and transrectal ultrasound (TRUS) showed a prostate volume of 59 ml, without any hypo-echoic lesions found. The PSA density was 0.19 ng/ml^2^. The need to perform MRI and biopsy: Based on PSA and PSA density, a biopsy and a pre-biopsy MRI are both indicated. MRI identified a suspicious lesion of approximately 10 mm in the left peripheral zone, wedge-shaped with substantial low ADC values, with a total PI-RADS assessment category score 4 (4/4/ +). Histopathology biopsy findings and treatment management: Biopsies revealed two positive cores out of two left-sided targeted cores and two positive cores out of five right-sided systematic cores, all with GS 3 + 4 with cribriform growth present. Right-sided systematic biopsies also detected GS 3 + 3 in two out-of-five cores. Patient underwent robot-assisted radical prostatectomy with pT2a, GS3 + 4 with cribriform pathology with clear resection margins (R0). Considerations: When clinical and MRI findings are concordant, the likelihood of clinically significant cancers is increased. Targeted and systematic biopsies are required. Use MRI as a roadmap to direct biopsy cores. Note that cribriform pattern is underestimated by MRI. Note also that systematic cores are often more likely to be positive for cancer in sextants adjacent to MRI target lesions, hence the recommendation for focal saturation biopsies for small suspicious lesions

It may be possible to deploy only MRI-directed targeted biopsies for men with larger PI-RADS 5 lesions (Fig. 5), resulting in reductions of the number of biopsy cores per patient, reserving combined systematic and targeted biopsies for smaller PI-RADS 4 lesions (Fig. 6) or heterogenous PI-RADS 3 lesions, to improve tumor grade classification and tumor volume estimations [20]. MRI-guided focal saturation biopsy where cores are placed in the MRI depicted target and in sextants adjacent is a viable alternative to combined systematic and targeted biopsy [20, 21], although the exact number and placement of biopsy cores is not yet well documented. In addition, a diffuse abnormality does not require targeted biopsy, as systematic cores alone will usually be enough. Note also that for patients with clinically obvious disease (i.e., very high PSA levels and a suspicious rectal examination), a bi-parametric MRI for biopsy planning and pelvic staging purposes is often enough.

In men with positive MRI, the prevalence of significant cancers increases with increasing PI-RADS category scores. While PI-RADS score 5 has the highest likelihood of having clinically significant prostate cancer, not every PI-RADS 5 lesions will harbor clinically significant disease (Fig. 5). False positive results may be due to misinterpretations of nodules of stromal benign prostatic hyperplasia within the central gland, due to large ISUP = 1 cancers or due to inflammation [22, 23]. Non-cancer causes of positive MRI decrease with higher PI-RADS scores. However, it is unsafe to assume that all PI-RADS 5 lesions are significant cancers; biopsy is always needed for confirmation.

## Golden rules for prostate MRI usage

The introduction of prostate MRI has substantially improved the accuracy of prostate cancer diagnosis, cancer grading and pre-treatment risk assessment. Following the current adoption of a pre-biopsy MRI in all men [2–4, 19], the radiological community has to deal with new challenges, such as improving quality of the entire diagnostic chain (Fig. 1) and increasing machine and manpower capacity. Implementation of this new diagnostic test in biopsy-naïve men needs the full attention of the radiological community. Within this emerging field, we need to commit to rules that guide physicians and patients to the optimal diagnostic work-up for identifying or excluding significant prostate cancer (Table 1).

**Fig. 7:**
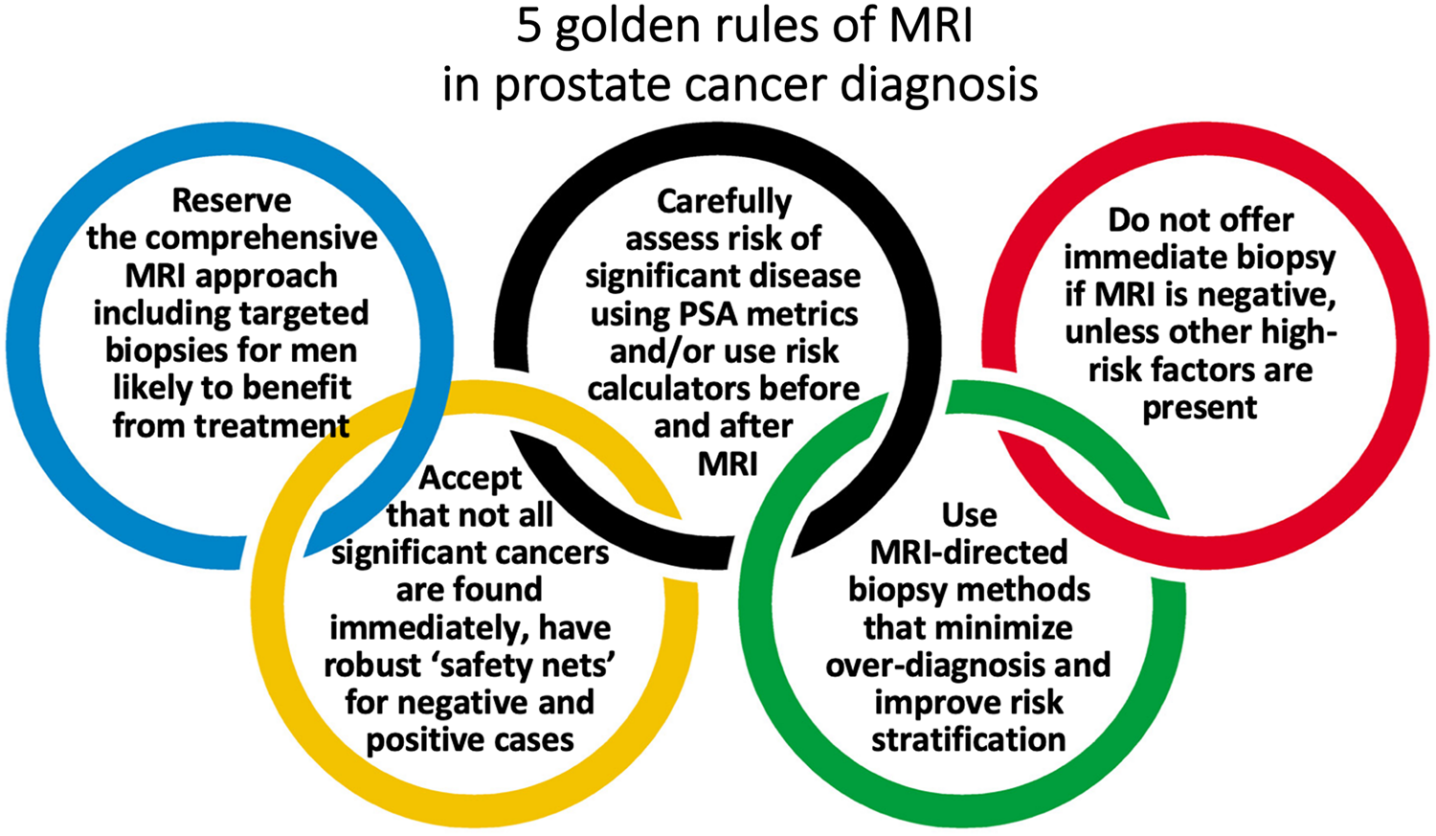
Five golden rules of MRI in prostate cancer diagnosis

The five golden rules of MRI for prostate cancer diagnosis (Fig. 7) are (1) reserve the comprehensive MRI approach including targeted biopsies for men likely to benefit from early detection and treatment of prostate cancer; (2) carefully assess risk of significant disease using PSA metrics and/or use risk calculators before and after MRI; (3) do not offer immediate biopsy if the MRI is negative, unless other high-risk factors are present; (4) accept that not all significant cancers are found immediately and have robust ‘safety nets’ for men with negative MRI scans who avoid immediate biopsy and for positive MRI patients with negative or non-explanatory histology; and (5) use MRI-directed biopsy methods that minimize overdiagnosis and improve risk stratification.
